# Supplementary material for: HIVprotI: an integrated web based platform for prediction and design of HIV proteins inhibitors
Source: J Cheminform. 2018 Mar 9;10:12. doi: 10.1186/s13321-018-0266-y (PMC5845081; doi:10.1186/s13321-018-0266-y)
Supplement: Supplementary file 2 — Additional file 2. Source code of HIVProtI web server. [file 13321_2018_266_MOESM2_ESM.zip › HIVprotI_Source-code/hivprotI/weka-3-6-6/doc/weka/experiment/DatabaseUtils.html]

DatabaseUtils


---


|  |  |  |
| --- | --- | --- |
| **Overview** \| **Package** \| **Class** \| **Tree** \| **Deprecated** \| **Index** \| **Help** \| **Weka's home** | |  |
| **PREV CLASS**   **NEXT CLASS** | **FRAMES**    **NO FRAMES**     **All Classes** |  |
| SUMMARY: NESTED \| FIELD \| CONSTR \| METHOD | DETAIL: FIELD \| CONSTR \| METHOD |  |


---


## weka.experiment Class DatabaseUtils

```
java.lang.Object
  weka.experiment.DatabaseUtils
```

**All Implemented Interfaces:**: java.io.Serializable, RevisionHandler

**Direct Known Subclasses:**: DatabaseConnection, DatabaseResultListener, DbUtils, InstanceQuery

---

``` public class DatabaseUtils extends java.lang.Object implements java.io.Serializable, RevisionHandler ```

DatabaseUtils provides utility functions for accessing the experiment
database. The jdbc
driver and database to be used default to "jdbc.idbDriver" and
"jdbc:idb=experiments.prp". These may be changed by creating
a java properties file called DatabaseUtils.props in user.home or
the current directory. eg:

```` ```
 jdbcDriver=jdbc.idbDriver
 jdbcURL=jdbc:idb=experiments.prp
``` ```` 

**Version:**
:   $Revision: 5240 $

**Author:**
:   Len Trigg (trigg@cs.waikato.ac.nz)

**See Also:**: Serialized Form

---

| **Field Summary** | |
| --- | --- |
| `static int` | `BOOL`             Type mapping for BOOL used for reading experiment results. |
| `static int` | `BYTE`             Type mapping for BYTE used for reading experiment results. |
| `static int` | `DATE`             Type mapping for DATE used for reading experiment results. |
| `static int` | `DOUBLE`             Type mapping for DOUBLE used for reading experiment results. |
| `static java.lang.String` | `EXP_INDEX_TABLE`             The name of the table containing the index to experiments. |
| `static java.lang.String` | `EXP_RESULT_COL`             The name of the column containing the results table name. |
| `static java.lang.String` | `EXP_RESULT_PREFIX`             The prefix for result table names. |
| `static java.lang.String` | `EXP_SETUP_COL`             The name of the column containing the experiment setup (parameters). |
| `static java.lang.String` | `EXP_TYPE_COL`             The name of the column containing the experiment type (ResultProducer). |
| `static int` | `FLOAT`             Type mapping for FLOAT used for reading experiment results. |
| `static int` | `INTEGER`             Type mapping for INTEGER used for reading experiment results. |
| `static int` | `LONG`             Type mapping for LONG used for reading experiment results. |
| `static java.lang.String` | `PROPERTY_FILE`             The name of the properties file. |
| `static int` | `SHORT`             Type mapping for SHORT used for reading experiment results. |
| `static int` | `STRING`             Type mapping for STRING used for reading experiment results. |
| `static int` | `TEXT`             Type mapping for TEXT used for reading, e.g., text blobs. |
| `static int` | `TIME`             Type mapping for TIME used for reading TIME columns. |


**Constructor Summary**

`DatabaseUtils()`
  
          Reads properties and sets up the database drivers.


| **Method Summary** | |
| --- | --- |
| `static java.lang.String` | `arrayToString(java.lang.Object[] array)`             Converts an array of objects to a string by inserting a space between each element. |
| `void` | `close()`             closes the m\_PreparedStatement to avoid memory leaks. |
| `void` | `close(java.sql.ResultSet rs)`             closes the ResultSet and the statement that generated the ResultSet to avoid memory leaks in JDBC drivers - in contrast to the JDBC specs, a lot of JDBC drives don't clean up correctly. |
| `void` | `connectToDatabase()`             Opens a connection to the database. |
| `void` | `createExperimentIndex()`             Attempts to create the experiment index table. |
| `java.lang.String` | `createExperimentIndexEntry(ResultProducer rp)`             Attempts to insert a results entry for the table into the experiment index. |
| `java.lang.String` | `createResultsTable(ResultProducer rp, java.lang.String tableName)`             Creates a results table for the supplied result producer. |
| `java.lang.String` | `databaseURLTipText()`             Returns the tip text for this property. |
| `java.lang.String` | `debugTipText()`             Returns the tip text for this property. |
| `void` | `disconnectFromDatabase()`             Closes the connection to the database. |
| `boolean` | `execute(java.lang.String query)`             Executes a SQL query. |
| `boolean` | `experimentIndexExists()`             Returns true if the experiment index exists. |
| `java.lang.String` | `getDatabaseURL()`             Get the value of DatabaseURL. |
| `boolean` | `getDebug()`             Gets whether there should be printed some debugging output to stderr or not. |
| `java.lang.String` | `getKeywords()`             Returns the currently stored keywords (as comma-separated list). |
| `java.lang.String` | `getKeywordsMaskChar()`             Returns the currently set mask character. |
| `java.lang.String` | `getPassword()`             Get the database password. |
| `java.lang.Object[]` | `getResultFromTable(java.lang.String tableName, ResultProducer rp, java.lang.Object[] key)`             Executes a database query to extract a result for the supplied key from the database. |
| `java.sql.ResultSet` | `getResultSet()`             Gets the results generated by a previous query. |
| `java.lang.String` | `getResultsTableName(ResultProducer rp)`             Gets the name of the experiment table that stores results from a particular ResultProducer. |
| `java.lang.String` | `getRevision()`             Returns the revision string. |
| `int` | `getSupportedCursorScrollType()`             Returns the type of scrolling that the cursor supports, -1 if not supported or not connected. |
| `java.lang.String` | `getUsername()`             Get the database username. |
| `boolean` | `isConnected()`             Returns true if a database connection is active. |
| `boolean` | `isCursorScrollable()`             Checks whether cursors are scrollable in general, false otherwise (also if not connected). |
| `boolean` | `isCursorScrollSensitive()`             Returns whether the cursors only support forward movement or are scroll sensitive (with ResultSet.CONCUR\_READ\_ONLY concurrency). |
| `boolean` | `isKeyword(java.lang.String s)`             Checks whether the given string is a reserved keyword. |
| `java.lang.String` | `maskKeyword(java.lang.String s)`             If the given string is a keyword, then the mask character will be appended and returned. |
| `java.lang.String` | `passwordTipText()`             Returns the tip text for this property. |
| `static java.lang.String` | `processKeyString(java.lang.String s)`             processes the string in such a way that it can be stored in the database, i.e., it changes backslashes into slashes and doubles single quotes. |
| `void` | `putResultInTable(java.lang.String tableName, ResultProducer rp, java.lang.Object[] key, java.lang.Object[] result)`             Executes a database query to insert a result for the supplied key into the database. |
| `java.sql.ResultSet` | `select(java.lang.String query)`             Executes a SQL SELECT query that returns a ResultSet. |
| `void` | `setDatabaseURL(java.lang.String newDatabaseURL)`             Set the value of DatabaseURL. |
| `void` | `setDebug(boolean d)`             Sets whether there should be printed some debugging output to stderr or not. |
| `void` | `setKeywords(java.lang.String value)`             Sets the keywords (comma-separated list) to use. |
| `void` | `setKeywordsMaskChar(java.lang.String value)`             Sets the mask character to append to table or attribute names that are a reserved keyword. |
| `void` | `setPassword(java.lang.String password)`             Set the database password. |
| `void` | `setUsername(java.lang.String username)`             Set the database username. |
| `boolean` | `tableExists(java.lang.String tableName)`             Checks that a given table exists. |
| `int` | `translateDBColumnType(java.lang.String type)`             translates the column data type string to an integer value that indicates which data type / get()-Method to use in order to retrieve values from the database (see DatabaseUtils.Properties, InstanceQuery()). |
| `static java.lang.String` | `typeName(int type)`             Returns the name associated with a SQL type. |
| `int` | `update(java.lang.String query)`             Executes a SQL DDL query or an INSERT, DELETE or UPDATE. |
| `java.lang.String` | `usernameTipText()`             Returns the tip text for this property. |

**Methods inherited from class java.lang.Object**

`equals, getClass, hashCode, notify, notifyAll, toString, wait, wait, wait`

**Field Detail**

### EXP\_INDEX\_TABLE

```
public static final java.lang.String EXP_INDEX_TABLE
```

:   The name of the table containing the index to experiments.

    **See Also:**: Constant Field Values

---


### EXP\_TYPE\_COL

```
public static final java.lang.String EXP_TYPE_COL
```

:   The name of the column containing the experiment type (ResultProducer).

    **See Also:**: Constant Field Values

---


### EXP\_SETUP\_COL

```
public static final java.lang.String EXP_SETUP_COL
```

:   The name of the column containing the experiment setup (parameters).

    **See Also:**: Constant Field Values

---


### EXP\_RESULT\_COL

```
public static final java.lang.String EXP_RESULT_COL
```

:   The name of the column containing the results table name.

    **See Also:**: Constant Field Values

---


### EXP\_RESULT\_PREFIX

```
public static final java.lang.String EXP_RESULT_PREFIX
```

:   The prefix for result table names.

    **See Also:**: Constant Field Values

---


### PROPERTY\_FILE

```
public static final java.lang.String PROPERTY_FILE
```

:   The name of the properties file.

    **See Also:**: Constant Field Values

---


### STRING

```
public static final int STRING
```

:   Type mapping for STRING used for reading experiment results.

    **See Also:**: Constant Field Values

---


### BOOL

```
public static final int BOOL
```

:   Type mapping for BOOL used for reading experiment results.

    **See Also:**: Constant Field Values

---


### DOUBLE

```
public static final int DOUBLE
```

:   Type mapping for DOUBLE used for reading experiment results.

    **See Also:**: Constant Field Values

---


### BYTE

```
public static final int BYTE
```

:   Type mapping for BYTE used for reading experiment results.

    **See Also:**: Constant Field Values

---


### SHORT

```
public static final int SHORT
```

:   Type mapping for SHORT used for reading experiment results.

    **See Also:**: Constant Field Values

---


### INTEGER

```
public static final int INTEGER
```

:   Type mapping for INTEGER used for reading experiment results.

    **See Also:**: Constant Field Values

---


### LONG

```
public static final int LONG
```

:   Type mapping for LONG used for reading experiment results.

    **See Also:**: Constant Field Values

---


### FLOAT

```
public static final int FLOAT
```

:   Type mapping for FLOAT used for reading experiment results.

    **See Also:**: Constant Field Values

---


### DATE

```
public static final int DATE
```

:   Type mapping for DATE used for reading experiment results.

    **See Also:**: Constant Field Values

---


### TEXT

```
public static final int TEXT
```

:   Type mapping for TEXT used for reading, e.g., text blobs.

    **See Also:**: Constant Field Values

---


### TIME

```
public static final int TIME
```

:   Type mapping for TIME used for reading TIME columns.

    **See Also:**: Constant Field Values


**Constructor Detail**

### DatabaseUtils

```
public DatabaseUtils()
              throws java.lang.Exception
```

:   Reads properties and sets up the database drivers.

    **Throws:**: `java.lang.Exception` - if an error occurs


**Method Detail**

### translateDBColumnType

```
public int translateDBColumnType(java.lang.String type)
```

:   translates the column data type string to an integer value that indicates
    which data type / get()-Method to use in order to retrieve values from the
    database (see DatabaseUtils.Properties, InstanceQuery()). Blanks in the type
    are replaced with underscores "\_", since Java property names can't contain blanks.

    :   **Parameters:**: `type` - the column type as retrieved with java.sql.MetaData.getColumnTypeName(int) **Returns:**: an integer value that indicates which data type / get()-Method to use in order to retrieve values from the

---


### arrayToString

```
public static java.lang.String arrayToString(java.lang.Object[] array)
```

:   Converts an array of objects to a string by inserting a space
    between each element. Null elements are printed as ?

    :   **Parameters:**: `array` - the array of objects **Returns:**: a value of type 'String'

---


### typeName

```
public static java.lang.String typeName(int type)
```

:   Returns the name associated with a SQL type.

    :   **Parameters:**: `type` - the SQL type **Returns:**: the name of the type

---


### databaseURLTipText

```
public java.lang.String databaseURLTipText()
```

:   Returns the tip text for this property.

    :   **Returns:**: tip text for this property suitable for displaying in the explorer/experimenter gui

---


### getDatabaseURL

```
public java.lang.String getDatabaseURL()
```

:   Get the value of DatabaseURL.

    :   **Returns:**: Value of DatabaseURL.

---


### setDatabaseURL

```
public void setDatabaseURL(java.lang.String newDatabaseURL)
```

:   Set the value of DatabaseURL.

    :   **Parameters:**: `newDatabaseURL` - Value to assign to DatabaseURL.

---


### debugTipText

```
public java.lang.String debugTipText()
```

:   Returns the tip text for this property.

    :   **Returns:**: tip text for this property suitable for displaying in the explorer/experimenter gui

---


### setDebug

```
public void setDebug(boolean d)
```

:   Sets whether there should be printed some debugging output to stderr or not.

    :   **Parameters:**: `d` - true if output should be printed

---


### getDebug

```
public boolean getDebug()
```

:   Gets whether there should be printed some debugging output to stderr or not.

    :   **Returns:**: true if output should be printed

---


### usernameTipText

```
public java.lang.String usernameTipText()
```

:   Returns the tip text for this property.

    :   **Returns:**: tip text for this property suitable for displaying in the explorer/experimenter gui

---


### setUsername

```
public void setUsername(java.lang.String username)
```

:   Set the database username.

    :   **Parameters:**: `username` - Username for Database.

---


### getUsername

```
public java.lang.String getUsername()
```

:   Get the database username.

    :   **Returns:**: Database username

---


### passwordTipText

```
public java.lang.String passwordTipText()
```

:   Returns the tip text for this property.

    :   **Returns:**: tip text for this property suitable for displaying in the explorer/experimenter gui

---


### setPassword

```
public void setPassword(java.lang.String password)
```

:   Set the database password.

    :   **Parameters:**: `password` - Password for Database.

---


### getPassword

```
public java.lang.String getPassword()
```

:   Get the database password.

    :   **Returns:**: Password for Database.

---


### connectToDatabase

```
public void connectToDatabase()
                       throws java.lang.Exception
```

:   Opens a connection to the database.

    :   **Throws:**: `java.lang.Exception` - if an error occurs

---


### disconnectFromDatabase

```
public void disconnectFromDatabase()
                            throws java.lang.Exception
```

:   Closes the connection to the database.

    :   **Throws:**: `java.lang.Exception` - if an error occurs

---


### isConnected

```
public boolean isConnected()
```

:   Returns true if a database connection is active.

    :   **Returns:**: a value of type 'boolean'

---


### isCursorScrollSensitive

```
public boolean isCursorScrollSensitive()
```

:   Returns whether the cursors only support forward movement or are
    scroll sensitive (with ResultSet.CONCUR\_READ\_ONLY concurrency).
    Returns always false if not connected

    :   **Returns:**: true if connected and the cursor is scroll-sensitive **See Also:**: `ResultSet.TYPE_SCROLL_SENSITIVE`, `ResultSet.TYPE_FORWARD_ONLY`, `ResultSet.CONCUR_READ_ONLY`

---


### isCursorScrollable

```
public boolean isCursorScrollable()
```

:   Checks whether cursors are scrollable in general, false otherwise
    (also if not connected).

    :   **Returns:**: true if scrollable and connected **See Also:**: `getSupportedCursorScrollType()`

---


### getSupportedCursorScrollType

```
public int getSupportedCursorScrollType()
```

:   Returns the type of scrolling that the cursor supports, -1 if not
    supported or not connected. Checks first for TYPE\_SCROLL\_SENSITIVE
    and then for TYPE\_SCROLL\_INSENSITIVE. In both cases CONCUR\_READ\_ONLY
    as concurrency is used.

    :   **Returns:**: the scroll type, or -1 if not connected or no scrolling supported **See Also:**: `ResultSet.TYPE_SCROLL_SENSITIVE`, `ResultSet.TYPE_SCROLL_INSENSITIVE`

---


### execute

```
public boolean execute(java.lang.String query)
                throws java.sql.SQLException
```

:   Executes a SQL query. Caller must clean up manually with
    `close()`.

    :   **Parameters:**: `query` - the SQL query **Returns:**: true if the query generated results **Throws:**: `java.sql.SQLException` - if an error occurs **See Also:**: `close()`

---


### getResultSet

```
public java.sql.ResultSet getResultSet()
                                throws java.sql.SQLException
```

:   Gets the results generated by a previous query. Caller must clean up
    manually with `close(ResultSet)`. Returns null if object has
    been deserialized.

    :   **Returns:**: the result set. **Throws:**: `java.sql.SQLException` - if an error occurs **See Also:**: `close(ResultSet)`

---


### update

```
public int update(java.lang.String query)
           throws java.sql.SQLException
```

:   Executes a SQL DDL query or an INSERT, DELETE or UPDATE.

    :   **Parameters:**: `query` - the SQL DDL query **Returns:**: the number of affected rows **Throws:**: `java.sql.SQLException` - if an error occurs

---


### select

```
public java.sql.ResultSet select(java.lang.String query)
                          throws java.sql.SQLException
```

:   Executes a SQL SELECT query that returns a ResultSet. Note: the ResultSet
    object must be closed by the caller.

    :   **Parameters:**: `query` - the SQL query **Returns:**: the generated ResultSet **Throws:**: `java.sql.SQLException` - if an error occurs

---


### close

```
public void close(java.sql.ResultSet rs)
```

:   closes the ResultSet and the statement that generated the ResultSet to
    avoid memory leaks in JDBC drivers - in contrast to the JDBC specs, a lot
    of JDBC drives don't clean up correctly.

    :   **Parameters:**: `rs` - the ResultSet to clean up

---


### close

```
public void close()
```

:   closes the m\_PreparedStatement to avoid memory leaks.

---


### tableExists

```
public boolean tableExists(java.lang.String tableName)
                    throws java.lang.Exception
```

:   Checks that a given table exists.

    :   **Parameters:**: `tableName` - the name of the table to look for. **Returns:**: true if the table exists. **Throws:**: `java.lang.Exception` - if an error occurs.

---


### processKeyString

```
public static java.lang.String processKeyString(java.lang.String s)
```

:   processes the string in such a way that it can be stored in the
    database, i.e., it changes backslashes into slashes and doubles single
    quotes.

    :   **Parameters:**: `s` - the string to work on **Returns:**: the processed string

---


### getResultFromTable

```
public java.lang.Object[] getResultFromTable(java.lang.String tableName,
                                             ResultProducer rp,
                                             java.lang.Object[] key)
                                      throws java.lang.Exception
```

:   Executes a database query to extract a result for the supplied key
    from the database.

    :   **Parameters:**: `tableName` - the name of the table where the result is stored: `rp` - the ResultProducer who will generate the result if required: `key` - the key for the result **Returns:**: true if the result with that key is in the database already **Throws:**: `java.lang.Exception` - if an error occurs

---


### putResultInTable

```
public void putResultInTable(java.lang.String tableName,
                             ResultProducer rp,
                             java.lang.Object[] key,
                             java.lang.Object[] result)
                      throws java.lang.Exception
```

:   Executes a database query to insert a result for the supplied key
    into the database.

    :   **Parameters:**: `tableName` - the name of the table where the result is stored: `rp` - the ResultProducer who will generate the result if required: `key` - the key for the result: `result` - the result to store **Throws:**: `java.lang.Exception` - if an error occurs

---


### experimentIndexExists

```
public boolean experimentIndexExists()
                              throws java.lang.Exception
```

:   Returns true if the experiment index exists.

    :   **Returns:**: true if the index exists **Throws:**: `java.lang.Exception` - if an error occurs

---


### createExperimentIndex

```
public void createExperimentIndex()
                           throws java.lang.Exception
```

:   Attempts to create the experiment index table.

    :   **Throws:**: `java.lang.Exception` - if an error occurs.

---


### createExperimentIndexEntry

```
public java.lang.String createExperimentIndexEntry(ResultProducer rp)
                                            throws java.lang.Exception
```

:   Attempts to insert a results entry for the table into the
    experiment index.

    :   **Parameters:**: `rp` - the ResultProducer generating the results **Returns:**: the name of the created results table **Throws:**: `java.lang.Exception` - if an error occurs.

---


### getResultsTableName

```
public java.lang.String getResultsTableName(ResultProducer rp)
                                     throws java.lang.Exception
```

:   Gets the name of the experiment table that stores results from a
    particular ResultProducer.

    :   **Parameters:**: `rp` - the ResultProducer **Returns:**: the name of the table where the results for this ResultProducer are stored, or null if there is no table for this ResultProducer. **Throws:**: `java.lang.Exception` - if an error occurs

---


### createResultsTable

```
public java.lang.String createResultsTable(ResultProducer rp,
                                           java.lang.String tableName)
                                    throws java.lang.Exception
```

:   Creates a results table for the supplied result producer.

    :   **Parameters:**: `rp` - the ResultProducer generating the results: `tableName` - the name of the resultsTable **Returns:**: the name of the created results table **Throws:**: `java.lang.Exception` - if an error occurs.

---


### setKeywords

```
public void setKeywords(java.lang.String value)
```

:   Sets the keywords (comma-separated list) to use.

    :   **Parameters:**: `value` - the list of keywords

---


### getKeywords

```
public java.lang.String getKeywords()
```

:   Returns the currently stored keywords (as comma-separated list).

    :   **Returns:**: the list of keywords

---


### setKeywordsMaskChar

```
public void setKeywordsMaskChar(java.lang.String value)
```

:   Sets the mask character to append to table or attribute names that
    are a reserved keyword.

    :   **Parameters:**: `value` - the new character

---


### getKeywordsMaskChar

```
public java.lang.String getKeywordsMaskChar()
```

:   Returns the currently set mask character.

    :   **Returns:**: the character

---


### isKeyword

```
public boolean isKeyword(java.lang.String s)
```

:   Checks whether the given string is a reserved keyword.

    :   **Parameters:**: `s` - the string to check **Returns:**: true if the string is a keyword **See Also:**: `m_Keywords`

---


### maskKeyword

```
public java.lang.String maskKeyword(java.lang.String s)
```

:   If the given string is a keyword, then the mask character will be
    appended and returned. Otherwise, the same string will be returned
    unchanged.

    :   **Parameters:**: `s` - the string to check **Returns:**: the potentially masked string **See Also:**: `m_KeywordsMaskChar`, `isKeyword(String)`

---


### getRevision

```
public java.lang.String getRevision()
```

:   Returns the revision string.

    :   **Specified by:**: `getRevision` in interface `RevisionHandler`
    :   **Returns:**: the revision


---


|  |  |  |
| --- | --- | --- |
| **Overview** \| **Package** \| **Class** \| **Tree** \| **Deprecated** \| **Index** \| **Help** \| **Weka's home** | |  |
| **PREV CLASS**   **NEXT CLASS** | **FRAMES**    **NO FRAMES**     **All Classes** |  |
| SUMMARY: NESTED \| FIELD \| CONSTR \| METHOD | DETAIL: FIELD \| CONSTR \| METHOD |  |


---
